# Expression Profiles of HOXC6 Predict the Survival of Glioblastoma Patients and Correlate with Cell Cycle

**DOI:** 10.1155/2022/8656865

**Published:** 2022-04-06

**Authors:** Zhen-Hang Li, Yue Ma, Yan Zhou, Zhen-Hua Huang

**Affiliations:** ^1^Department of Neurosurgery, Tianjin Huanhu Hospital, Tianjin, China; ^2^Clinical College of Neurology, Neurosurgery and Neurorehabilitation, Tianjin Medical University, Tianjin, China

## Abstract

The goal of this study was to investigate the homeobox (HOX) gene expression status and its prognostic value in glioblastoma multiforme (GBM) and to uncover the biological processes related to its expression. The prognostic value of HOX genes in GBM was systematically investigated by a genome-wide analysis of HOX gene expression profiles in GBM patient samples in The Cancer Genome Atlas (TCGA) project (microarray dataset) and validation datasets. Using the differentially expressed gene (DEG) analysis and a Cox regression model, we discovered that the HOXC6 could stratify patients into significantly different survival (*p* = 0.0012, log-rank test) groups in the training cohort. TCGA RNA-seq and GSE16011 datasets were used for validation. Multivariate Cox and stratification analysis indicated that HOXC6 was an independent prognostic factor after adjusting for other clinical covariates. Bioinformatic analysis suggested that the HOXC6 might be involved in the cell cycle-related biological processes and pathways that are well established in the context of glioblastoma tumorigenesis. We further explored the bioinformatic implications by gene set enrichment analysis (GSEA). Tumor cell biology experiments verified the role of HOXC6 in proliferation and cell cycle progression. In conclusion, HOXC6 might be a candidate biomarker gene for individual treatment optimization of glioblastoma. HOXC6 expression has a significant prognostic value and is related to the cell cycle process in glioblastoma.

## 1. Introduction

Brain gliomas can be categorized into low-grade glioma (grade I), lower-grade gliomas (LGG, grades II and III), and highly malignant glioblastoma multiforme (GBM, grade IV) [1]. The most common and aggressive form is GBM, which has a mean overall survival (OS) of 14.6 months and a 2-year survival rate of 26.5% with standard therapy [2]. The “integrated” [3] phenotypic and genotypic parameters for the central nervous system (CNS) tumor classification were introduced in the 2016 World Health Organization Classification of Tumors of the Central Nervous System [4], which emphasized the molecular impact on tumorigenesis and prognosis of glioma. The clinical characteristics of GBM that contribute to its dismal prognosis are aggressive growth, limited response to therapy, and inexorable recurrence [5]. The emergence of molecularly focused approaches to cancer has fundamentally changed the path to the diagnosis and treatment of malignancies. Histology is increasingly supplemented with molecular analysis, and these data subsequently inform therapeutic decision-making [6].

Homeobox (HOX) genes act as master regulators of morphogenesis and cell differentiation and participate in the maintenance of adult cellular identity [7]. Recently, the altered expression of HOX genes has been associated with multiple solid tumors, including colon, gastric, breast, bladder, lung, and prostate cancer; nasopharyngeal carcinoma; and childhood acute lymphoblastic leukemia [8–19]. Furthermore, it was also observed that the expression of the HOXC family genes was upregulated in most solid tumor types [20]. HOX genes exhibit tissue-specific functions and can promote tumorigenesis as a consequence of their gain- or loss-of-function mutations, which dysregulates growth and differentiation [21].

While a multitude of molecular prognostic markers have been proposed for GBM (e.g., [22]), prognostic values of the HOX gene expression have yet to be precisely elaborated. In this study, we demonstrated that HOXC6 could distinguish the clinical and molecular features of GBM. HOXC6 was of significant prognostic value in patients with glioblastoma in the Cancer Genome Atlas (TCGA) microarray dataset, and this finding was validated by additional public datasets. Bioinformatic analysis illustrated that HOXC6 expression was highly correlated with cell cycle-related processes, especially in the M phase. These results will likely provide a new paradigm to the GBM research and facilitate therapeutic decision-making.

## 2. Material and Methods

### 2.1. Datasets

The whole genome mRNA expression microarray data and corresponding clinical information (sex, age, isocitrate dehydrogenase (IDH) mutation status, O6-methylguanin-DNA-methyltransferase (MGMT) methylation status, chemotherapy status, radiotherapy status, and survival information) from the TCGA dataset (http://cancergenome.nih.gov/) were downloaded as a training set [23]. The TCGA dataset [24], which contains more than two petabytes of open access genomic data, helps researchers improve the prevention, diagnosis, and treatment of cancer. The whole genome mRNA expression RNA-seq data and corresponding clinical information from the TCGA dataset (http://cancergenome.nih.gov/) were downloaded as a validation set. GSE16011 (containing 155 GBM cases with available clinical data, http://www.ncbi.nlm.nih.gov/geo/query/acc.cgi?acc=GSE16011) [25] datasets were also obtained as validation sets.

### 2.2. Bioinformatic Analysis

For preliminary investigation into the functions of the genes highly correlated with the risk group, gene ontology (GO) biological process enrichment analysis, GO cellular components, and Kyoto Encyclopedia of Genes and Genomes (KEGG) pathway enrichment analysis were carried out using the R package “TCGAbiolinks” [26]. The cutoff criterion was FDR <0.01.

The GSEA (gene set enrichment analysis) was performed using Gene Set Enrichment Analysis v3.0 software downloaded from the Broad Institute (http://www.broadinstitute.org/gsea). The mRNA expression profile of GBM samples from the training dataset was analyzed by GSEA [27]. For GSEA, the gene expression was treated as a binary variable divided into low or high based on whether the gene expression was greater than the cutoff value.

### 2.3. Cell Culture and Transfection

The human glioblastoma cell lines LN229 and T98G were purchased from Beijing Beina Chuanglian Biotechnology Institute and cultured in DMEM containing 10% fetal bovine serum (FBS, Gibco, Invitrogen, CA, USA). siRNA targeting HOXC6 mRNA was produced by GenePharma (Suzhou, China). The HOXC6 mRNA overexpression plasmid (gene vector: GV657) was produced by GeneChem (Shanghai, China). The transfections were performed using Lipofectamine 2000 according to the manufacturer's instructions (Thermo Fisher Scientific).

### 2.4. RNA Extraction and Real-Time PCR

TRIzol reagent was used for RNA extraction, and the SureFireRT kit (06-104; Abgen) was used for RNA reverse transcription. Real-time PCR was conducted on a LightCycler 480 II (Roche). The sequences of the PCR primers were as follows: GAPDH forward, 5′-CAATGACCCCTTCATTGACC-3′ and reverse, 5′-GACAAGCTTCCCGTTCTCAG-3′, and HOXC6 forward, 5′- CACCGCCTATGATCCAGTGAGGCA-3′ and reverse, 5′-GCTGGAACTGAACACGACATTCTC-3′. The PCR conditions were 95 °C for 5 min, followed by 40 cycles at 95 °C for 10 s and 60 °C for 1 min. Relative quantity of the gene product was calculated by the 2−*ΔΔ*Ct method.

### 2.5. CCK-8 and Cell Cycle Analysis

Five thousand (5 × 10^3^) cells per well were seeded in 96-well plates and transfected with siRNA (siHOXC6 and siRNA scramble, siScr) or overexpression plasmid (oe-DNA3.1-HOXC6 and control, oe-DNA3.1). CCK-8 reagent (K009-500, ZETA) was added to the cells at 0, 24, 48, and 72 h after transfection, and the mixture was incubated for 30–60 min at room temperature. Optical density was measured at 450 nm on a molecular device microplate reader. For the cell cycle assay, glioblastoma cells were harvested 48 h after transfection and fixed with precooled 75% alcohol for 3 h. Subsequently, the cells were incubated with 0.5 ml of PI/RNase reagent (BD Biosciences, USA) at room temperature for 20 min and analyzed by flow cytometry.

### 2.6. Western Blot

Tissue and cell protein were promptly homogenized in a homogenization buffer containing 1 M TrisHCl pH 7.5, 1% Triton X-100, 1% Nonidet p-40 (NP-40), 10% sodium dodecyl sulfate (SDS), 0.5% sodium deoxycholate, 0.5 M EDTA, leupeptin 10 lg/mL, aprotinin 10 lg/mL, and 1 mMPMSF and then centrifuged to collect the supernatant liquid. Protein concentrations were determined with a Bio-Rad protein assay (Bio-Rad, Hercules, CA, USA). The total cellular protein extracts were separated by sodium dodecyl sulfate–polyacrylamide gel electrophoresis (SDS-PAGE) and transferred to polyvinylidene difluoride filter (PVDF) membranes (Millipore, Bedford, MA). After the membranes were blocked in 5% nonfat milk in TBST (150 mMNaCl, 20 mMTris, and 0.05% Tween 20) for 2 hours, they were incubated with the primary antibodies overnight at 4 °C. Then, the membranes were washed with TBST for three times, 10 minutes each, and then horseradish-peroxidase-linked IgG as the secondary antibodies for 2 hours at room temperature. The membrane was developed using the ECL detection systems. The experiments were carried out in three separate occasions.

### 2.7. Antibodies

The antibodies used for Western blot analysis were as follows: antihuman HOXC6 monoclonal antibody (Affinity Biosciences, China), antihuman minichromosome maintenance complex component 2 (MCM2) monoclonal antibody (Affinity Biosciences, China), antihuman cyclin E polyclonal antibody (Affinity Biosciences, China), E2F transcription factor 1 (E2F1) monoclonal antibody (Affinity Biosciences, China), antihuman proliferating cell nuclear antigen (PCNA) monoclonal antibody (Affinity Biosciences, China), antihuman cyclin D1 polyclonal antibody (Santa Cruz Biotechnology, USA), antihuman cyclin dependent kinase 2 (CDK2) polyclonal antibody (Santa Cruz Biotechnology, USA), and antihuman glyceraldehyde 3-phosphate dehydrogenase (GAPDH) polyclonal antibody (Santa Cruz Biotechnology, USA).

### 2.8. Statistical Analysis

The Shapiro-Wilks normality test was performed to validate the gene expression profile in the training set using the R package “mclust” [28]. The cutoff was determined by the mean expression for genes in a normally distributed population. For genes that were not normally distributed, the expression pattern was divided into two Gaussian distributions, and the cutoff was determined by the intersection between the two Gaussian distributions. The package “limma” (Affymetrix) [29] was used to identify the differentially expressed genes (DEGs) between GBM tissue and normal brain tissue (NBT). The correlations of gene expression were analyzed using R (Pearson correlation). The chi-square test was performed with Yate's correction for continuity using R. The two-tailed Student *t*-test was performed to compare two groups of numerical values using R. The analysis of variance (ANOVA) with false discovery correction (FDR) was performed to identify genes that were differentially expressed among gliomas of increasing grade using R. The chi-squared test and Fisher's exact test were used to compare the frequencies between groups in R. All differences were considered statistically significant at the level of *p* < 0.05.

OS was defined as the period from the first operation to death or last follow-up. The differences in OS between patients expressing high levels of HOXC6 and those with low expression were estimated using the Kaplan–Meier method and two-sided log-rank test in GraphPad Prism Version 7.00. The univariate and multivariate Cox proportional hazards regression analyses were performed to assess the contribution of the genes and clinicopathologic variables to survival prediction.

## 3. Results

### 3.1. Identification of the Prognostic HOX Gene from the Training Cohort

**Figure 1:**
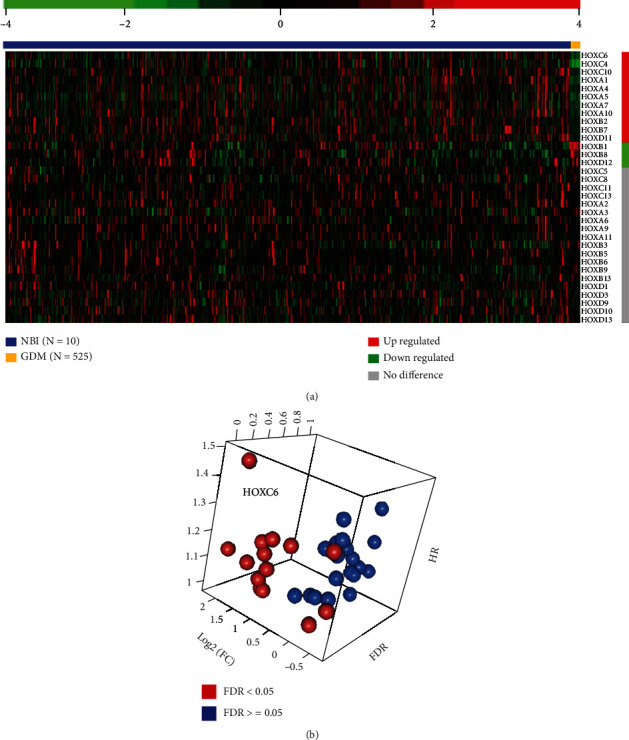
(a) Gene expression analysis of 33 HOX genes from 535 samples of TCGA microarray dataset identified 14 differentially expressed genes. The columns represent samples, and rows represent the *Z* score of the gene expression value. Color bar: red indicates high relative expression levels, whereas green indicates low levels. Vertical bar: red indicates upregulated genes, green indicates downregulated genes, and gray indicates no difference. Horizontal bar: blue indicates GBM tissues, whereas yellow indicates normal brain tissues (NBT). (b) Distribution of HOX genes based on differential expression gene analysis and univariate Cox regression analysis. HR: hazard ratio; Log2(FC): log2 value of fold change; FDR: false discovery rate of *p* value in the differential expression gene analysis. Blue balls indicate genes that are not differentially expressed, whereas red balls indicate genes that are differentially expressed. The left top red ball indicates HOXC6.

We first extracted data on 33 HOX genes from the gene expression profiles of 535 samples in the TCGA microarray dataset. To compare the expression profiles between normal and GBM tissues, we performed differentially expressed gene (DEG) analysis and found that 11 genes were upregulated and 3 genes were downregulated, while the other 19 genes showed no significant differences (Figure 1(a)).

We performed the Shapiro-Wilks normality test on the HOX genes and found that none of the 33 genes distributed as one Gaussian distribution. Thus, we divided the expression patterns to fit into two Gaussian distributions, one with high-expression values and the other with low-expression values. The same approach was used to define the cutoff value for validation groups. To evaluate the relationship between HOX gene expression and prognostic value, the patients were stratified into low or high-expression groups according to the cutoff value for each HOX gene. The univariate Cox regression analysis confirmed that 7 HOX genes were associated with the survival of patients with glioblastoma (*p* < 0.05). After sorting by the hazard ratio (HR) value combined with the DEG test, we discovered that HOXC6 had the highest HR value, with a log2 (fold change) of 1.931 (Figure 1(b)). We also noted that high HOXC6 expression was correlated with age (*p* = 0.001; *χ*2 test) and IDH1 mutation status (*p* < 0.001; *χ*2 test) (data not shown).

### 3.2. HOXC6 Expression Profile in Gliomas

**Figure 2:**
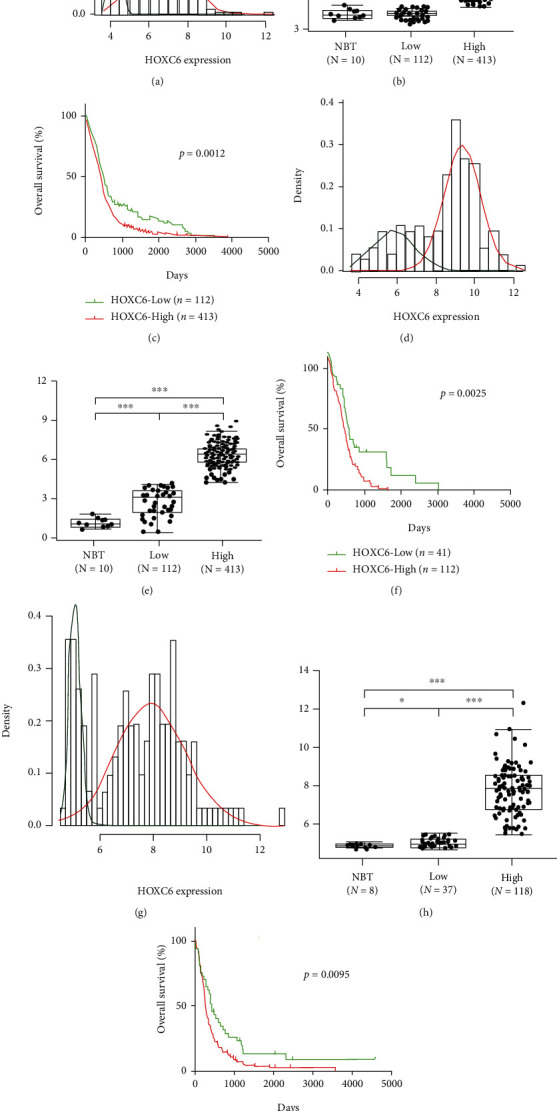
HOXC6 expression profile predicted the survival of GBM patients in different datasets. (a-c) TCGA microarray dataset. (a) Gaussian mixture modeling to identify bimodal expression patterns. (b) Expression profile of HOXC6 in normal brain tissue (NBT), low-expression group (low) and high-expression group (high) was analyzed using the Student *t*-test. (c) Kaplan–Meier survival analyses based on the cutoff of HOXC6 expression value in the TCGA microarray dataset. (d-f) TCGA RNA-seq dataset. (d) Gaussian mixture modeling to identify bimodal expression patterns. (e) Expression profile of HOXC6 among normal brain tissue (NBT), low-expression group (low), and high-expression group (high) was analyzed using the Student *t*-test. (f) Kaplan–Meier survival analyses based on the cutoff of HOXC6 expression value in the TCGA RNA-seq dataset. (g-i) GSE16011 dataset. (g) Gaussian mixture modeling to identify bimodal expression patterns. (h) Expression profile of HOXC6 among normal brain tissue (NBT), low-expression group (low), and high-expression group (high) was analyzed using the Student *t*-test. (i) Kaplan–Meier survival analyses based on the cutoff of HOXC6 expression value in the GSE16011 dataset. NS. *p* ≥ 0.05; ∗*p* < 0.05; ∗∗*p* < 0.01; ∗∗∗*p* < 0.001.

We explored the HOXC6 expression pattern in the training and validation datasets and noticed that HOXC6 expression was highly upregulated in GBM tissues compared to normal brain tissues (data not shown). HOXC6 expression was absent or low in normal brain tissues and pilocytic astrocytoma (PA, grade I), high in GBM, and intermediate in LGG (data not shown). The HOXC6 expression in the training dataset, TCGA RNA-seq dataset, and GSE16011 dataset was distributed as two Gaussian distributions (Figures 2(a), 2(d), and 2(g)). High and low groups were defined according to the cutoff value of each dataset, and there was a significant difference in the expression level between them. The expression of HOXC6 in the high group was significantly higher than that in normal brain tissues in all three datasets, while the low group showed no or only a slight difference compared with normal brain tissues in the training (*p* > 0.05, *t*-test) and GSE16011 (*p* = 0.033, *t*-test) datasets.

### 3.3. HOXC6 Is an Independent Prognostic Factor in the Training Cohort

To explore whether HOXC6 expression was related to the GBM patient survival, we first analyzed the training dataset containing 525 cases. The samples were dichotomized into either high (*n* = 413) or low (*n* = 112) subgroups according to the HOXC6 expression cutoff value. Our analyses demonstrated that the high HOXC6 expression was a potent independent marker for predicting worse OS in the TCGA microarray dataset (*p* = 0.0012, log-rank test; Figure 2(c)).

### 3.4. Independence of HOXC6 from Other Clinical Variables and Molecular Features

We then stratified the patients based on several clinicopathologic factors, including age, sex, chemotherapy, radiotherapy, transcriptional subtype [30], MGMT methylation status, and isocitrate dehydrogenase (IDH)1 mutation status. The univariate and multivariate Cox regression analyses were performed using age, sex, chemotherapy, radiotherapy, and the HOXC6 expression level as covariables. The results from the Cox analyses revealed that HOXC6 was significantly associated with OS when adjusted for age, sex, chemotherapy, and radiotherapy (Table 1). The cutoff value in the subgroups was the same as the cutoff value in the training set. We observed that the patients expressing high levels of HOXC6 had significantly shorter OS than those expressing low levels of HOXC6 in corresponding cohorts. The HOXC6 expression classified 320 male patients into a high-expression group (*n* = 252) and a low-expression group (*n* = 68) with a significantly different OS (*p* = 0.0039, log-rank test). According to the age, the GBM patients could be stratified into a younger patient group (age <60, *n* = 273) and an older patient group (age ≥60, *n* = 252). There was a significant difference in the OS between the high-expression group (*n* = 199) and the low-expression group (*n* = 74) in the younger patient group (*p* = 0.0473, log-rank test).

We also noted that high HOXC6 expression was highly correlated with IDH1 mutation status (*p* < 0.001; *χ*2 test). Furthermore, we investigated whether the predictive power of HOXC6 was independent of IDH1 mutation status using multivariate Cox regression analysis. The multivariate Cox regression analysis suggested that HOXC6 was statistically significantly associated with survival (*p* = 0.0142) when adjusted for IDH mutation status, indicating that the predictive ability of HOXC6 was independent of IDH1 mutation status for the survival of patients with GBM. In the unfavorable subgroup defined by IDH1 wild-type, the patients with high expression of HOXC6 (*n* = 309) had shorter OS (*p* = 0.0191, log-rank test) than the low-expression group (*n* = 67). In the favorable subgroup defined by methylated MGMT status, the patients with high expression of HOXC6 (*n* = 130) still had shorter OS (*p* = 0.0391, log-rank test) than the low-expression group (*n* = 27).

We compared the expression level of HOXC6 across four glioblastoma subtypes (classical, mesenchymal, neural, and proneural) and found that only the proneural subtype had a significant heterogeneity in the expression levels of HOXC6 among four glioblastoma subtypes (*p* = 0.003, *t*-test). Further, the Kaplan–Meier survival analysis demonstrated that, apart from the proneural subtype, the high-expression group (*n* = 116) in the classical subtype showed a worse prognosis (*p* = 0.0330, log-rank test) than the low-expression group (*n* = 39). There was a significant difference (*p* = 3.9e − 05, log-rank test) in the proneural subtype between the high-expression group (*n* = 83) and the low-expression group (*n* = 24).

We analyzed the prognostic power of chemotherapy and radiotherapy in combination with HOXC6 expression and other clinical covariates by multivariate Cox regression analysis (Table 1). The Kaplan–Meier survival analysis demonstrated that, in patients who underwent chemotherapy, the high-expression group (*n* = 287) showed a worse prognosis (*p* = 0.0188, log-rank test) than the low-expression group (*n* = 84). A similar result was obtained in the radiotherapy subgroup; the high-expression group (*n* = 301) showed a worse prognosis (*p* = 0.0096, log-rank test) than the low-expression group (*n* = 90). These results indicated that the HOXC6 classification accurately identified patients with poor prognosis irrespective of these clinicopathologic risk factors.

### 3.5. Validation of HOXC6 as a Prognostic Marker in Other Cohorts

We analyzed the relationship between HOXC6 gene expression and patient survival information in the TCGA RNA-seq cohort. The patients were stratified into groups based on the HOXC6 cutoff value: 112 cases were in the high-expression group, and 41 cases were in the low-expression group. Log-rank test results showed significant differences (*p* = 0.0025, log-rank test; Figure 2(f)) between the high-expression group and low-expression group in a Kaplan–Meier survival plot. We obtained similar results by analyzing the GSE16011 cohort (there were 159 GBMs in the cohort, and OS in 4 cases was not available). The high-expression group (*n* = 118) showed a worse prognosis (*p* = 0.0095, log-rank test; Figure 2(i)) than the low-expression group (*n* = 37). These results indicated that the HOXC6 expression profile could independently predict clinical outcomes of GBM cases across multiple datasets and platforms.

### 3.6. In Silico Functional Analysis of HOXC6

To gain new insights into the function of HOXC6, we performed in silico functional analysis to reveal the potential biological roles of HOXC6 in GBM. For this purpose, all genes in the training set were compared with HOXC6 expression using pairwise Pearson correlations (r value ranged from −0.2675 to 0.7839, of which the genes with *r* value >0.4 are all HOX genes), with absolute *r* value ≥0.2 and *p* < 0.01 being considered significant (*n* = 945). A dichotomization based on the HOXC6 expression cutoff was carried out for the DEG analysis of all genes in the training dataset and identified the genes with FDR *p* value <0.05 (*n* = 1256).

**Figure 3:**
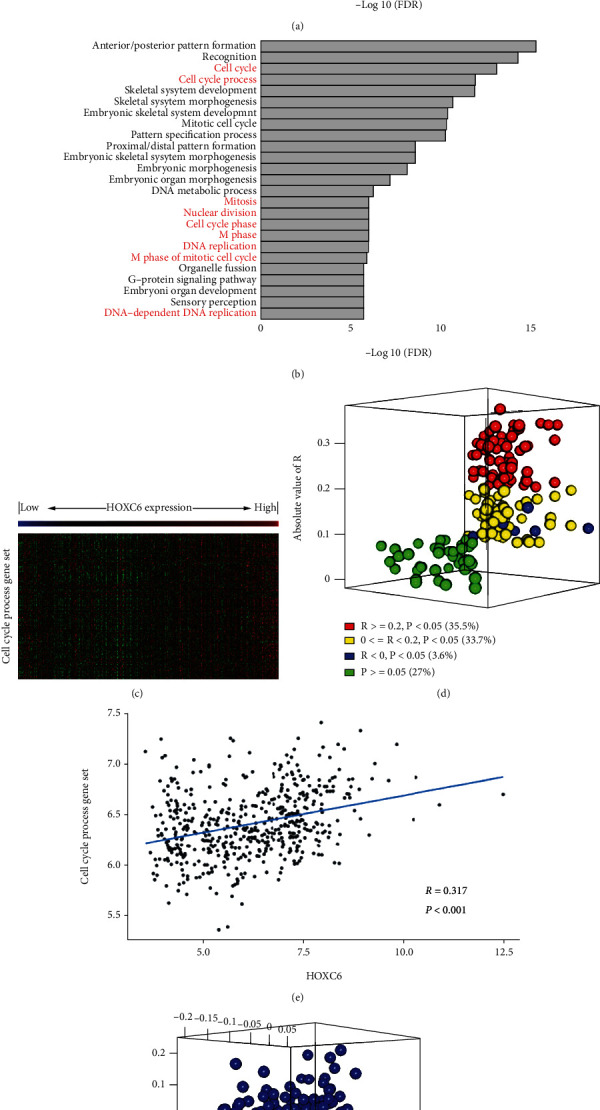
GO biological process analysis. (a) Top 25 GO terms enriched in genes highly correlated with HOXC6. (b) Top 25 GO terms enriched in DEGs between the high-expression group and the low-expression group of HOXC6. (c) Heat map showing the expression levels of genes from the cell cycle process gene set in relation to the HOXC6 expression levels. (d) 3D plot showing the relative Pearson correlation *r* value of the genes from the cell cycle process gene set. (e) Correlation plot showing the Pearson correlation between HOXC6 expression and the mean expression value of the genes from the cell cycle process gene set. (f) 3D plot showing the principal component analysis between HOXC6 and the genes from the cell cycle process gene set. PC: principal component.

Following the GO enrichment analysis, we found that the functions of highly correlated genes and DEGs shared great similarity. The results showed that most of the highly enriched biological processes were related to the cell cycle (Figures 3(a) and 3(b)). The cell cycle process gained the most significance in both scenarios. Thus, we further investigated the relationship between cell cycle process genes with HOXC6. A heat map (a graphical representation of data where the individual values contained in a matrix are represented as colors) showed that the relative gene expression from the cell cycle process gene set increased with the HOXC6 expression level (Figure 3(c)). The Pearson correlation *r* value of the genes from the cell cycle process gene set was compared with HOXC6 and visualized as a 3D plot (Figure 3(d)). When the mean expression value of the cell cycle process genes was analyzed and plotted against HOXC6 expression (Figure 3(e)), it showed a high correlation (*r* = 0.317, *p* < 0.001). Further, the principal component analysis (PCA) was carried out (Figure 3(f)) with the expression values of cell cycle process genes and HOXC6, and the results showed that HOXC6 was among the cell cycle process genes in the PCA.

**Figure 4:**
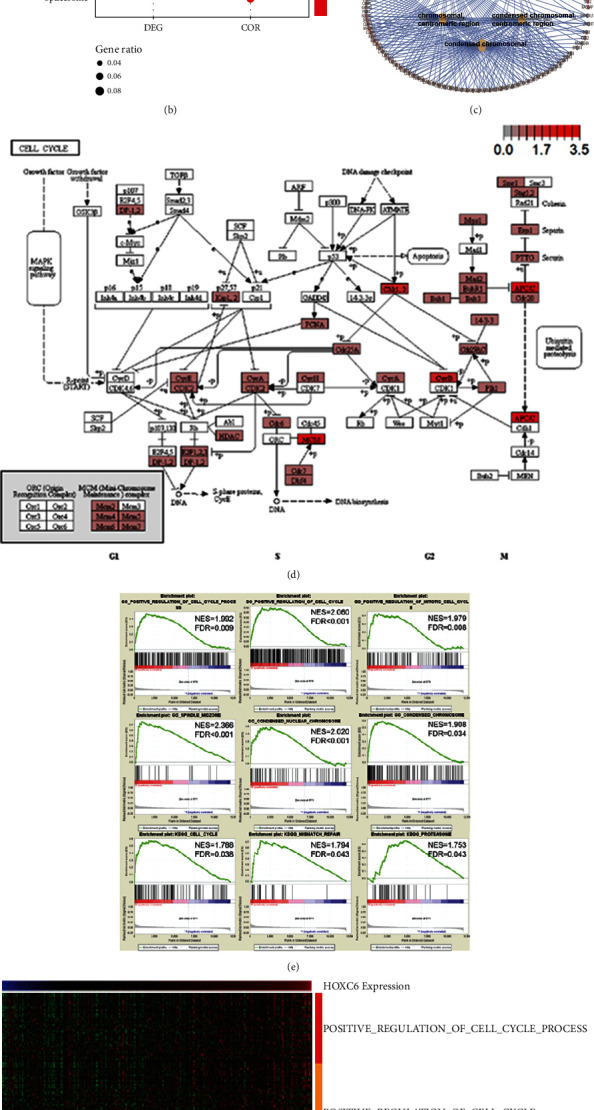
GO cellular component analysis and KEGG pathway enrichment analysis. (a) Merged top 5 GO terms enriched either in DEGs or genes highly correlated with HOXC6. (b) Merged top 5 KEGG pathways enriched either in DEGs or genes highly correlated with HOXC6. (c) Yellow circles represent the top 5 GO cellular components enriched in genes highly correlated with HOXC6, whereas brown circles represent the enriched genes. (d) KEGG pathway analysis based on genes highly correlated with HOXC6. Colored rectangles represent the highly correlated genes, and the color bar indicates the log2-fold change of genes between the high-expression group and the low-expression group of HOXC6. GSEA and heat map represents GSEA results. (e) GSEA was performed in patients sorted by HOXC6 expression value. Above: biological process, genes associated with positive regulation of cell cycle process, cell cycle, and mitotic cell cycle are enriched in patients with high levels of HOXC6 expression; center: cellular component, genes associated with spindle midzone, condensed nuclear chromosome, and condensed chromosome are enriched in patients with high levels of HOXC6 expression; below: KEGG pathway, genes associated with cell cycle, mismatch repair, and proteasome pathways are enriched in patients with high levels of HOXC6 expression. The horizontal bar in graded color from red to blue represents the rank ordering of patients based on decreasing levels of HOXC6 expression. Vertical black lines represent the projection of individual genes constituting relevant gene sets. (f) Heat map showing enriched genes from the GSEA gene sets of Figure 4(e) in combination with the HOXC6 expression value. DEG: differentially expressed gene; COR: highly correlated gene.

Cellular component enrichment analysis and KEGG pathway analysis illustrated that both DEGs and genes highly correlated with HOXC6 were enriched in cell cycle-related components and pathways (Figures 4(a) and 4(b)). The highly correlated genes enriched in cell cycle-related cellular components are shown in Figure 4(c). The enriched cell cycle pathway is shown in Figure 4(d). Further GSEA confirmed that cases with high HOXC6 expression exhibited high enrichment scores in cell cycle-related gene sets (Figure 4(e)). The genes enriched in GSEA were plotted in the heat map showing their relative expression pattern with HOXC6 expression (Figure 4(f)).

### 3.7. Biological Functions of HOXC6 in Human Glioblastoma Cells

**Figure 5:**
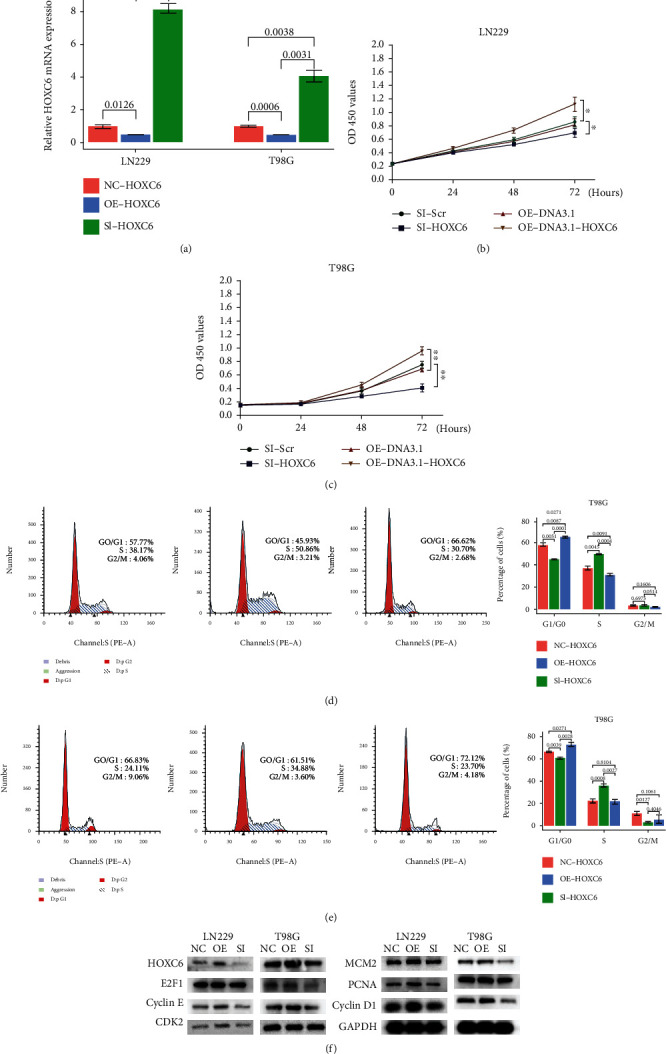
Tumor cell biology experiments of HOXC6. (a) Expression of HOXC6 after transfection of siRNA or overexpression plasmid measured by PCR. (b) CCK-8 assay to evaluate the effect of HOXC6 knockdown or overexpression on cell proliferation in LN229 cells. ∗*p* < 0.05, ∗∗*p* < 0.01. (c) CCK-8 assay to evaluate the effect of HOXC6 knockdown or overexpression on cell proliferation in T98G cells. From left to right: NC, OE, and SI. (d) Flow cytometry analysis to evaluate the effects of HOXC6 on cell cycle progression in LN229 cells. (e) Flow cytometry analysis to evaluate the effects of HOXC6 on cell cycle progression in T98G cells. From left to right: NC, OE, and SI. (f) Western blot analysis to evaluate the effects of HOXC6 on expression level of cell cycle related proteins.

To explore the functions of HOXC6 in glioblastoma, LN229 and T98G cells were transfected with siRNAs or overexpression plasmids. The transfection significantly reduced or increased the mRNA levels as anticipated (Figure 5(a)). The CCK-8 assay showed that the transfection with siHOXC6 significantly reduced proliferation of the cells and the transfection with oe-DNA3.1-HOXC6 significantly promoted proliferation (Figures 5(b) and 5(c)). Cell cycle assays showed that the knockdown of HOXC6 induced cell cycle arrest and downregulation of DNA replication, while the overexpression of HOXC6 promoted the cell cycle progression (Figures 5(d) and 5(e)). As expected, the overexpression of knockdown of HOXC6 resulted in correspondent expression level changes of cell cycle–related proteins like E2F1, Cyclin E, CDK2, MCM2, PCNA, and Cyclin D1 (Figure 5(f)). These results suggest that HOXC6 could promote malignant phenotypes of glioblastoma cells in vitro.

## 4. Discussion

GBM is characterized by heterogeneity, suggesting that a variety of gene products may play a role in regulating their behavior and the ultimate outcome. GBM molecular classification is still in the early stage, and there is no general consensus on the GBM subtypes. Several studies [25, 31] have provided high-resolution images of the GBM molecular landscape and revealed significant changes that might contribute to the pathogenesis and biology of the disease. The dependence of the tumor on specific molecular pathways that are adapted to direct translation into the clinic has not yet been demonstrated.

We investigated the HOX gene expression in GBM, its prognostic value, and relationship with other pathologic parameters and discovered that HOXC6 might be useful as a potential biological marker. HOXC6 expression did not show a normal distribution in a large number of patients (e.g., TCGA datasets, GSE16011 dataset), of which approximately 70%~80% had increased levels of HOXC6 expression. We further investigated the prognostic value of HOXC6 in combination with clinical parameters and relevant markers of GBMs. Our study demonstrated that HOXC6 was not only a potent independent prognostic marker but also had the potential to refine the molecular classification of GBM in combination with other well-established markers. The HOXC6 expression profile identified subgroups with worse prognosis within the poor prognosis group identified by the IDH mutation status and the good prognosis group identified by the MGMT methylation status. Several recent studies emphasized the importance of using molecular markers to predict GBM prognosis and make an informed decision on potential adjuvant therapy [32–34]. Finally, we demonstrated that HOXC6 was an effective survival prognostic factor when compared with other factors, such as chemotherapy and radiotherapy, after adjustment for other clinical variables by multivariate survival analysis.

Until now, HOXC6 has mainly been known as a transcription factor (TF) during embryogenesis and neuronal differentiation [35]. It has been reported that HOXC6 directly regulates the expression of bone morphogenetic protein 7 (BMP7), fibroblast growth factor receptor 2 (FGFR2), insulin-like growth factor binding protein 3 (IGFBP-3), and platelet-derived growth factor receptor *α* (PDGFRA) in prostate cells and indirectly affects the Notch and Wnt signaling pathways. The transcriptional network regulated by HOXC6 plays crucial roles in the proliferation, survival, and metastasis of prostate cancer cells [13]. HOXC6 RNA has been shown to be a potential diagnostic marker for prostate cancer as part of a multigene panel [15] and has prognostic significance in gastric cancer [16], prostate cancer [17], breast cancer [18], and childhood acute lymphoblastic leukemia [19]. However, the exact mechanism by which HOXC6 regulates glioma tumorigenesis is still unclear. The results of GO analysis demonstrated that the genes involved in cell cycle-related biological processes and cellular components made up a large proportion of those highly correlated with HOXC6 or the differentially expressed genes (DEGs) between the high HOXC6 expression group and the low HOXC6 expression group. KEGG pathway analysis further revealed that these genes were commonly annotated as members of cell cycle–related pathways. GSEA validated the findings of GO and KEGG analyses, which demonstrated that HOXC6 could play a role in regulating the cell cycle-related processes in GBM. Finally, the tumor cell biology experiments further reinforced the validation. Genes correlated with HOXC6 were enriched in the cell cycle pathway, and they were predominantly localized in the minichromosome maintenance (MCM) complex. MCM protein was shown to be an important marker for estimating tumor proliferation and was a useful adjunct to the routinely used proliferation markers for glioblastoma diagnosis [36]. The strong correlation between HOXC6 and the MCM complex suggested that HOXC6 might be a candidate predictive marker for the radiosensitivity of GBM.

## 5. Conclusions

By evaluating its prognostic value, performing an in-depth bioinformatics analysis, and verifying with the tumor cell biology experiments, we showed that HOXC6 could predict the survival of GBM patients and that its expression correlated with the cell cycle-related genes. The HOXC6 expression profile may be useful in individual therapeutic decision-making either by itself or in combination with other clinicopathologic factors. We anticipate that the present study will enhance our understanding in the landscape of GBM tumorigenesis and facilitate future drug design, clinical trials, and patient-specific cell therapy.

## Figures and Tables

**Table 1:** Cox regression analysis of HOXC6 and other characteristics in GBM.

Variables	TCGA microarray Univariate		TCGA microarray Multivariate		TCGA RNA-seq Multivariate		GSE16011 Multivariate	
	HR	*p*	HR	*p*	HR	*p*	HR	*p*
HOXC6	1.47	0.0013	1.27	0.0481	1.82	0.0074	1.49	0.0486
Age	1.64	<0.0001	1.52	<0.0001	1.26	0.0899	1.19	0.1156
Sex	1.16	0.1223	1.16	0.1322	0.94	0.7655	1.07	0.7198
Chemotherapy	0.41	<0.0001	0.68	0.0168	0.43	0.0831	0.52	0.0982
Radiotherapy	0.35	<0.0001	0.52	0.0001	0.18	0.0004	0.42	0.0002

Hazard ratio for age variable: risk per 20 years. HR: hazard ratio; univariate: univariate Cox regression; multivariate: multivariate Cox regression; NA: not acquired.

## Data Availability

The preprocessed normalized expression data and clinical data of 525 GBM Microarray (training cohort) and 153 GBM RNA-seq samples belong to the TCGA dataset, retrieved from the website http://cancergenome.nih.gov/. The normalized expression data and clinical data of the GSE16011 dataset comprised of 155 GBM samples were retrieved from the Gene Expression Omnibus website.
